# Circular RNA-based therapeutics in ischemic stroke: emerging targets for neuroprotection

**DOI:** 10.1097/MS9.0000000000005619

**Published:** 2026-09-02

**Authors:** Sabeen Tahir, Rejina Chhetri, Janu Chhetri, Marina KC

**Affiliations:** a Federal Medical and Dental College, Islamabad, Pakistan; b Nepalgunj Medical College, Kohalpur, Banke, Nepal; c Department of medicine, Parbat provincial hospital, Badagaoon Sadak, Kushma, Nepal

*Dear Editor,*

Ischemic stroke remains one of the leading causes of death and long-term disability worldwide. According to the Global Burden of Disease Study 2021, an estimated 11.9 million people experienced a first-ever stroke in 2021, with 65.3% of all incident strokes being ischemic, underscoring its substantial global health burden^[^1^]^. Despite major advances in intravenous thrombolysis and mechanical thrombectomy, many patients remain ineligible for these interventions because of narrow therapeutic windows, while others continue to experience significant neurological deficits despite successful reperfusion. These limitations highlight the need for adjunctive neuroprotective strategies capable of minimizing secondary brain injury. In this context, circular RNAs (circRNAs) have recently emerged as promising therapeutic candidates owing to their remarkable stability, tissue-specific expression, and ability to regulate multiple molecular pathways involved in ischemic stroke.

The circRNAs are endogenous non-coding RNA molecules characterized by a covalently closed-loop structure that confers remarkable stability and resistance to degradation. Beyond serving as potential biomarkers, circRNAs regulate gene expression by acting as microRNA sponges, interacting with RNA-binding proteins, influencing transcription, and, in some cases, encoding functional peptides. Through these mechanisms, they modulate several pathological processes implicated in ischemic stroke, including neuronal apoptosis, oxidative stress, neuroinflammation, angiogenesis, and blood–brain barrier dysfunction. Their ability to influence multiple pathways simultaneously has generated increasing interest in circRNAs as promising therapeutic targets for limiting secondary brain injury and promoting neurological recovery after ischemic stroke^[^2^]^.

Recent experimental studies have reported encouraging results for circRNA-based therapies in ischemic stroke. In one study, circDLGAP4 reduced blood–brain barrier damage and improved neurological outcomes by limiting endothelial-to-mesenchymal transition after cerebral ischemia^[^3^]^. Another study demonstrated that extracellular vesicle-derived circSCMH1 enhanced neural plasticity and functional recovery in both rodent and non-human primate models of stroke, highlighting its potential for future clinical translation^[^4^]^. Although these findings are limited to preclinical models, they provide important proof-of-concept that targeting circRNAs may complement existing reperfusion therapies.

Despite these encouraging findings, several barriers must be overcome before circRNA-based therapies can be translated into clinical practice. Efficient delivery across the blood–brain barrier remains a major challenge, while concerns regarding off-target effects, long-term safety, and large-scale manufacturing require further investigation. In addition, most of the available evidence comes from preclinical studies, highlighting the need for well-designed clinical trials to establish the efficacy and safety of circRNA-based therapies in patients with ischemic stroke^[^2^]^.

In conclusion, future research should focus on developing reliable delivery systems, validating therapeutic targets in human studies, and determining how circRNA-based therapies can be integrated with current standards of stroke care. Addressing these challenges will be essential for translating the promising findings from experimental models into meaningful clinical benefits.

As our understanding of circRNA biology continues to expand, these molecules may offer a novel approach to neuroprotection by targeting multiple pathological pathways simultaneously. Although clinical application remains a future goal, continued translational research may pave the way for circRNA-based therapies to complement existing treatment strategies and improve outcomes for patients with ischemic stroke.

## Data Availability

No datasets were generated or analyzed during the preparation of this Letter to the Editor.

## Research registration unique identification number (UIN)

Not applicable for a Letter to the Editor, as per the journal.
